# Translational Quantitative Proteomic Assay for Bacteriophages: A New Frontier in Phage Pharmaceutical Development

**DOI:** 10.64898/2026.05.27.728049

**Published:** 2026-05-29

**Authors:** Thomas D. Nguyen, Connor E. Gould, Jacob T. Sanborn, Joshua Tutin, Yan Pan, Haoran Gao, Donna Ruszaj, Devin Agevine, Johnathan Bussa, Daniel Atakora, Liang Chen, Dwayne R. Roach, Troy D. Wood, Nicholas M. Smith

**Affiliations:** 1 Division of Clinical and Translational Therapeutics, School of Pharmacy & Pharmaceutical Sciences, University at Buffalo, Buffalo, New York, USA; 2 Center for Integrated Global Biomedical Sciences, Department of Pharmacy Practice, School of Pharmacy & Pharmaceutical Sciences, University at Buffalo, Buffalo, New York, USA; 3 Department of Pharmaceutical Sciences, School of Pharmacy & Pharmaceutical Sciences, University at Buffalo, Buffalo, New York, USA; 4 Department of Biology, San Diego State University, San Diego, California, USA; 5 Department of Chemistry, University at Buffalo, Buffalo, New York, USA

**Keywords:** Bacteriophage therapy, liquid chromatography-tandem mass spectrometry, signature peptide, phage quantitation, pharmacokinetics, *Pseudomonas aeruginosa*

## Abstract

Accurate quantitation of therapeutic bacteriophages (**phages**) remains a challenge for clinical development. Plaque-based enumeration is the current standard but is laborious, host-dependent, and variable, particularly when distinguishing individual phages in cocktails. Targeted mass spectrometry of virion structural proteins offers an orthogonal, structure-based approach amenable to reproducible and scalable phage quantitation. Here, we describe a targeted proteomic liquid chromatography-tandem mass spectrometry (**LC-MS/MS**) assay for host-independent quantitation of the *Pseudomonas aeruginosa* podovirus LUZ19. Proteomic characterization was performed on an LTQ Orbitrap XL to assess sequence coverage and select surrogate peptide candidates based on specificity and sensitivity. High-resolution peptide mapping identified multiple structural proteins of LUZ19 and provided 55% sequence coverage for the major head protein (YP_001671977.1). Fifteen peptides were detected and evaluated, from which the tryptic peptide EVAELDGQELAR was selected based on abundance, stability, and chromatographic performance. Quantitative analysis was conducted on a QTRAP 7500+ using optimized multiple reaction monitoring transitions for targeted peptide detection. Back-calculated concentrations met accuracy criteria across a validated range of 0.008 to 80 pg/mL, with bias spanning −8.2 to 8.2%, intra-day precision ranging from 0.5 to 9.8%, and inter-day precision ranging from 6.3 to 9.7%. Peptide concentrations from digested lysate samples were related to phage concentrations determined by double layer agar assay, yielding an estimated three copies of the major head protein per virion.

## Importance

Bacteriophages are the most abundant biological entities on the planet and represent a promising therapeutic class for combating drug-resistant bacterial infections. Realizing the clinical potential of bacteriophage therapy requires analytical methods capable of meeting the standards of modern drug development. Targeted mass spectrometry offers unmatched specificity and resolution for precise quantitation of individual bacteriophages within complex biological samples, a capability that conventional enumeration methods cannot match. Only one prior study has applied mass spectrometry to bacteriophage quantitation, using a well-characterized model bacteriophage at a single concentration without calibration or a validated analytical range. Using *Pseudomonas aeruginosa* podovirus LUZ19, we present the first targeted mass spectrometry-based bacteriophage quantitation assay developed and validated following FDA bioanalytical guidance. This work establishes a rigorous analytical foundation that moves bacteriophage therapy closer to the standards required for informed dose selection, candidate evaluation, and clinical development.

## Introduction

As antimicrobial resistance (**AMR**) continues to escalate into a global health crisis, conventional antibiotics are increasingly ineffective against a growing number of multidrug-resistant (**MDR**) and extensively drug-resistant (**XDR**) pathogens.[1, 2] This alarming trend has reignited interest in bacteriophages (**phages**) as a therapeutic alternative to combat AMR.[3, 4] Lytic phages, also known as virulent phages, are viruses that infect bacterial cells and replicate via the lytic cycle, wherein the infected bacterium is lysed to release newly formed phage progeny. However, the unique ability of phages to replicate in the presence of susceptible bacteria presents distinct challenges for both pharmacokinetic (**PK**) and pharmacodynamic (**PD**) analyses, particularly where distinguishing exogenously administered phages from endogenously replicated progeny phage can confound accurate quantitation and interpretation of phage exposure-response relationships during treatment.[5]

Currently, the double layer agar (**DLA**) assay remains the gold standard for phage enumeration, enabling quantitation of infectious phage particles within a solution.[6-8] The DLA assay remains a valuable reference method, as it selectively quantifies phages capable of completing a full lytic cycle under defined conditions. While broadly applicable, the assay must be optimized for each specific phage-host pair, and results can be highly variable without rigorous standardization.[6, 9] Even under standardized conditions, plaque enumeration is subject to significant variability arising from the Poisson distribution inherent to count data, establishing an irreducible analytical floor that persists independent of operator proficiency or procedural consistency.[9, 10] While pragmatic for demonstrating therapeutic efficacy, this approach does not allow for the resolution of individual phage concentrations, which is critical for PK analyses that support cocktail design, dose selection, and identification of target concentrations. Moreover, the assay is labor-intensive, low-throughput, and susceptible to operator bias, making it impractical for high-throughput preclinical workflows or future clinical implementation.

Quantitative polymerase chain reaction (**qPCR**) assays have been developed for high-throughput phage enumeration by measuring genome copy number, and have been proposed as a scalable analytical approach for clinical trial implementation.[9, 11-15] While highly sensitive and adaptable to complex matrices, qPCR does not differentiate between infectious and non-infectious particles, and genome copy number does not reliably reflect functional titer, often resulting in overestimation of the biologically active phage population.[9, 12, 16] In the context of PK/PD analyses, this distinction can be consequential, as phage therapy is governed by a dynamic predator-prey relationship in which phage replication, bactericidal activity, and resistance emergence are inextricably linked. Overestimated phage concentrations may therefore misguide dose selection, obscure true exposure-response relationships, and confound the pharmacodynamic indices necessary to characterize bactericidal activity over the course of treatment.[17] This consideration applies equally to droplet digital PCR (**ddPCR**), which addresses amplification efficiency variability but shares the same scope limitation with respect to particle viability. Reported ddPCR-derived phage concentrations have been consistently higher than PFU counts, reinforcing that amplification-based methods reflect a complementary but distinct quantity from infectious particle concentration. Additionally, as phages replicate and undergo genetic drift over the course of treatment, mutations in the targeted genomic region may progressively affect quantitation accuracy, a consideration that is difficult to detect without orthogonal measurement.[18] In a clinical setting where dosing decisions depend on accurate and reliable concentration measurements, the analytical scope of the measurement warrants careful consideration relative to the question being asked.

The translation of phage therapy into clinical practice hinges on the availability of robust analytical methods capable of supporting rigorous PK/PD characterization. Understanding exposure-response relationships is fundamental to phage candidate selection, dose selection, defining target concentrations, and characterizing the pharmacodynamic indices that link phage exposure to bactericidal activity and resistance suppression.[19] These requirements demand analytical methods that are precise, accurate, reproducible, and capable of resolving individual phage concentrations across biological matrices. Neither current plaque assay nor nucleic acid-based methods fully meet these analytical demands. Liquid chromatography and tandem mass spectrometry (**LC-MS/MS**), as the established bioanalytical platform for drug development, is uniquely positioned to address these gaps by providing the standardized, matrix-flexible, and regulatory-ready quantitation framework required for phage therapy clinical trial implementation.

**Figure 1:**
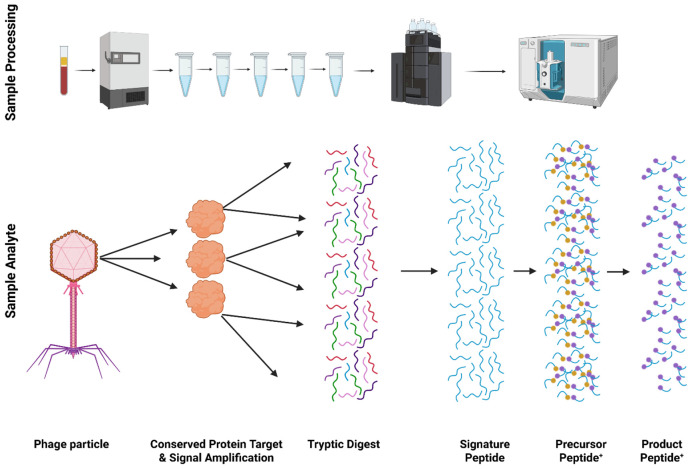
Conceptual overview of the targeted proteomic tandem mass spectrometry workflow for phage quantitation. Conserved structural proteins of the phage capsid serve as quantitative targets, leveraging fixed stoichiometry as an intrinsic signal amplification mechanism for reproducible, host-independent phage enumeration by tandem mass spectrometry.

LC-MS/MS using multiple reaction monitoring (**MRM**) offers high specificity, reproducibility, and high-throughput quantitation with multiplexing capabilities that are directly applicable to the demands of phage therapy development.[20-22] As the preferred platform for small molecule bioanalysis, LC-MS/MS operates within an established regulatory framework accepted by the FDA and EMA, providing a clear path toward validated clinical trial assays. Despite these compelling advantages, its application in phage enumeration remains extremely limited.[23-25] To date, only a single study has applied mass spectrometry to develop a quantitative platform for a well-characterized filamentous phage.[26] However, that study assessed only a single concentration and relied on prior knowledge of capsid protein copy numbers to infer phage particle counts. This approach would be challenging to extend to less well-characterized phages relevant to clinical application. The present study describes the development of a proteomic LC-MS/MS assay for the quantitation of a clinical *Pseudomonas aeruginosa* phage strain, LUZ19, leveraging conserved structural proteins as quantitative targets for host-independent phage quantitation (Figure 1).

## Results

### Proteomic Characterization of LUZ19 and Signature Peptide Selection

To prioritize candidates for quantitative method development, proteins were filtered based on structural annotation and abundance, resulting in four proteins identified from the NCBI FASTA database for further evaluation (Table 1). The identified proteins included major head and minor head proteins along with two hypothetical proteins. The major head protein yielded 55% sequence coverage from 15 identified peptides. The remaining proteins featured varying coverage coming from large tryptic peptides. The two hypothetical proteins YP_001671995.1 and YP_001671965.1 featured 46% and 2% sequence coverage derived from two and one tryptic peptides, respectively. Due to the high degree of sequence coverage and number of peptides identified, peptides derived from the major head protein YP_001671977.1 were identified as potential quantitative peptides. The tryptic peptide EVAELDGQELAR was selected as the quantitative peptide for the major head protein YP_001671977.1, identified with 8.72 ppm mass error for the precursor and an MS^2^ spectrum. The peptide was selected based on appropriate size for quantitation, high signal intensity post-digestion, and absence of easily modifiable amino acids.

### Copy Number Determination

**Figure 2:**
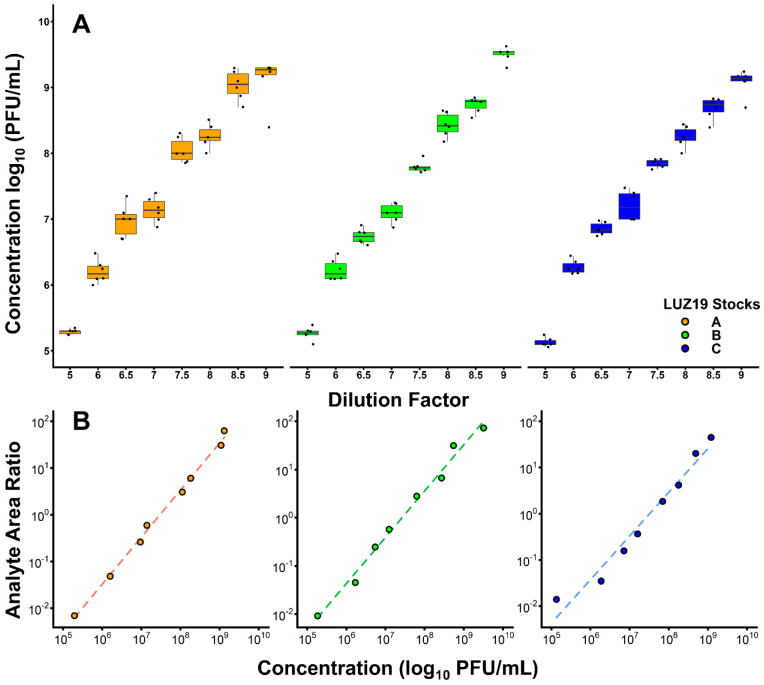
Quantitation of LUZ19 bacteriophage stocks by plaque assay and tandem mass spectrometry. (A) Variability of DLA-derived PFU/mL estimates across three independent LUZ19 phage stocks (A, B, and C) prepared at 10-fold dilutions spanning 10^5^ to 10^9^ PFU/mL. Box plots represent the distribution of sextuplicate measurements at each dilution level, with individual observations overlaid. Considerable inter- and intra-stock variability was observed across the concentration range, with CV ranging from 9.6% to 68.5%. Linear regression resulted in R^2^ values of 0.8510, 0.9092, and 0.9479 for stocks A, B, and C respectively. (B) Corresponding analysis of the same phage lysate stocks demonstrating the linear relationship between analyte area ratio and phage concentration for stocks A, B, and C on a log-log scale. Dashed lines represent linear regression fits for each individual stock, with R^2^ values of 0.9295, 0.9136, and 0.9135 for stocks A, B, and C respectively.

**Figure 3:**
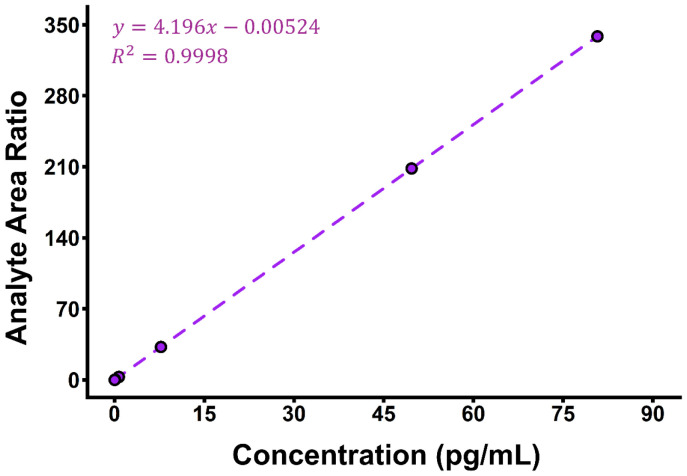
Calibration curve for the extended analog of the tryptic peptide EVAELDGQELAR. Analyte area ratio plotted against the concentration of the extended peptide analog EVAELDGQELARKFD across the calibration concentration range of 0.008 to 80 pg/mL. Following tryptic digestion, the tryptic peptide was quantified by tandem mass spectrometry. The dashed line represents the linear regression fit with a 1/x^2^ weighting factor, yielding a slope of 4.196, an intercept of −0.00524, and an R^2^ of 0.9998, demonstrating excellent linearity across the quantitative range. Analyte area ratios were calculated as peak area of analyte relative to the stable isotope-labeled internal standard.

The variability of the DLA method was assessed across three independent LUZ19 stocks (A, B, and C) prepared at concentrations ranging from 10^5^ to 10^9^ PFU/mL. Considerable variability in PFU/mL estimates was observed across stocks and dilution levels, with the coefficient of variation (CV) ranging from 9.6 to 68.5% and R^2^ values of 0.8510, 0.9092, and 0.9479 for stocks A, B, and C respectively (Figure 2A). Corresponding MS analysis of the same phage lysate stocks demonstrated strong linearity between peak area ratio and phage concentration across all three stocks, with R^2^ values of 0.9295, 0.9136, and 0.9135 for stocks A, B, and C respectively (Figure 2B). The calibration curve of the extended analog tryptic peptide EVAELDGQELARKFD demonstrated excellent linearity across the concentration range of 0.008 to 80 pg/mL with an R^2^ of 0.9998 (Figure 3).

**Figure 4:**
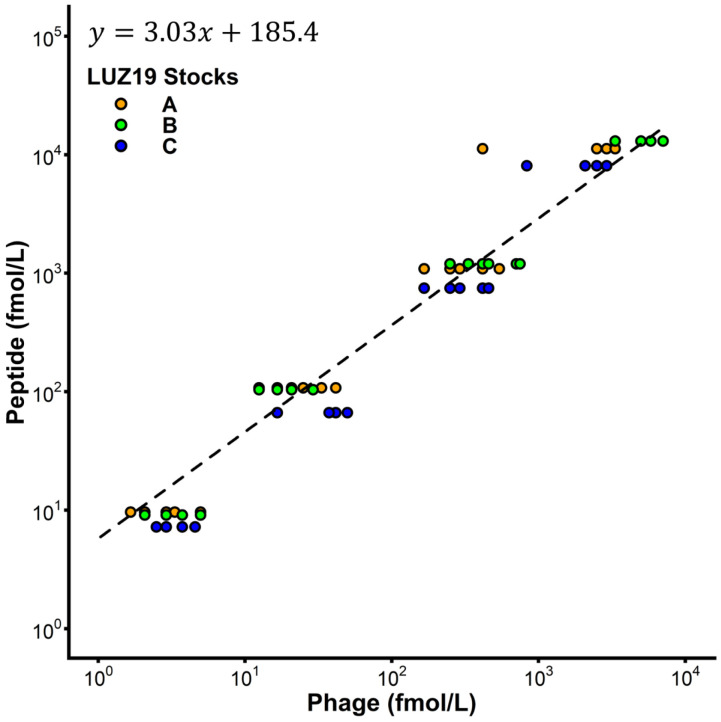
Deming regression relating peptide molarity to virion molarity for copy number determination. Peptide concentration (fmol/L) derived from LC-MS/MS measurements plotted against phage concentration (fmol/L) derived from DLA measurements across three independent LUZ19 stocks (A, B, and C) spanning 10^5^ to 10^9^ PFU/mL. Phage and peptide concentrations were converted to femtomolar units using Avogadro’s number and molecular weight of the tryptic peptide. The dashed line represents the Deming regression fit (y = 3.03x + 185.4) with an error ratio of 0.604, accounting for measurement error in both methods. The slope of 3.03 (95% CI: 2.61 – 3.75) represents the empirical estimate of major head protein copies per virion.

Peptide and phage molar concentrations were derived from mass spectrometry measurements and from plaque assay counts respectively, and expressed in femtomolar units for regression analysis. An empirically derived error ratio (λ = 0.604) was calculated from matched variance estimates across the concentration range, reflecting superior precision of the mass spectrometry relative to the DLA assay. Deming regression of the peptide molarity against the virion molarity yielded a slope of 3.03 (95% CI: 2.61 – 3.75), representing an empirical estimate of approximately three copies of the major head protein per virion (Figure 4), derived from absolute quantitation using a stable isotope-labeled standard. The intercept was not significantly different from zero (95% CI: −0.308 – 330.7 fmol/L), supporting a proportional relationship between peptide and virion molarity across the concentration range evaluated. The non-zero intercept estimate of 185.4 is attributable to upward bias in the peptide concentrations at the lowest phage concentration tested (10^5^ PFU/mL), which approached the lower limit of quantitation of the mass spectrometry assay, compounded by the high measurement variability of the plaque assay.

### Validation and Evaluation of Quantitative Performance

Method performance was evaluated using three independent analytical runs prepared on separate days. Back-calculated concentrations of calibration standards met acceptance criteria across the validated range of 0.008 to 80 pg/mL, with bias spanning −8.2 to 8.2% (Table 2). The tryptic peptide QC samples at 0.006, 0.4, and 40 pg/mL demonstrated intra-day precision ranging from 0.5% to 9.8% CV (Table 3) and inter-day precision ranging from 6.3% to 9.7% CV (Table 4), with back-calculated accuracy spanning −19.8% to 4.1% bias across each concentration level and run. Although one QC replicate exceeded the ± 15% acceptance threshold, greater than 67% of total QC samples and greater than 50% of replicates at each concentration level met acceptance criteria, consistent with FDA bioanalytical method validation guidance, confirming the reproducibility and accuracy of the assay.

**Figure 5:**
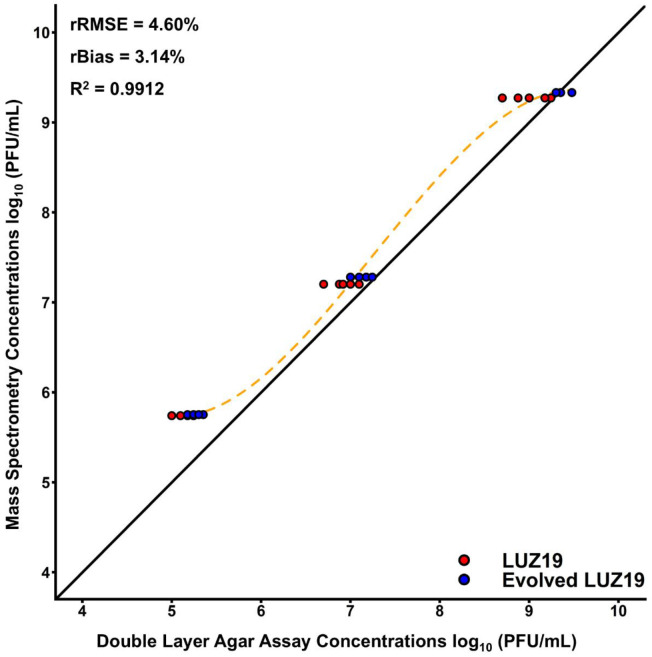
Agreement between LC-MS/MS and DLA-derived concentration estimates for unmodified and evolved LUZ19. Tandem mass spectrometry derived phage concentrations plotted against DLA-derived nominal concentrations on a log-log scale for unmodified LUZ19 (red) and evolved LUZ19 (blue) samples prepared at approximately 10^5^, 10^7^, and 10^9^ PFU/mL, analyzed in triplicate across three separate days. Overall method agreement was quantified by an rRMSE of 4.60% and rBias of 3.14% across all samples, with an R^2^ of 0.9912.

To evaluate the accuracy of the empirical copy number conversion factor, phage lysate QC samples from both the unmodified and evolved LUZ19 were prepared at approximately 10^5^, 10^7^, and 10^9^ PFU/mL and analyzed in triplicate across three separate days. Phage concentrations estimated by the mass spectrometry method using the empirical conversion factor were compared to nominal DLA-derived concentrations, yielding an rRMSE of 4.6% and rBias of 3.1% (Figure 5). The concordance of mass spectrometry estimated concentrations with DLA assay measurements for evolved LUZ19 confirmed conservation of the major head protein following directed evolution, demonstrating the robustness of the assay across phage variants.

### Phage Directed Evolution and Host Range Expansion

Nine clinical *Pseudomonas aeruginosa* isolates obtained from the CDC/FDAAR isolate bank were used for directed evolution and efficiency of plating assessment. At Day 0, LUZ19 demonstrated variable EOP across the clinical isolate panel relative to the reference strain PAO1, with several isolates showing limited or no susceptibility (Table 5). Following directed evolution, EOP increased for select clinical isolates, indicating successful host range expansion. The evolved LUZ19 demonstrated detectable lytic activity against isolates that were resistant or poorly susceptible to the unmodified phage at Day 0.

## Discussion

The development of phage therapy as a clinically viable treatment modality has long been constrained by the absence of analytical methods capable of meeting the quantitative standards expected in modern drug development. As a proof-of-concept for a single therapeutic phage, this work demonstrates that targeted tandem mass spectrometry can provide reproducible, host-independent quantitation through detection of a structural tryptic peptide, anchoring phage enumeration within the established paradigm of bioanalytical method development. Current approaches to phage quantitation each measure fundamentally distinct biological properties and carry inherent tradeoffs that reflect the respective analytical scope of each method. The DLA assay quantifies infectious particles capable of completing a lytic cycle under defined conditions, providing biologically meaningful information directly relevant to therapeutic efficacy, but is labor-intensive, highly variable, and unsuitable for high-throughput workflows. qPCR measures genome copy number with high sensitivity and throughput, capturing a distinct and complementary biological property, though it does not differentiate between infectious and non-infectious particles. This inability to differentiate infectious from non-infectious particles can be particularly consequential for exposure-response characterization in PK/PD analyses where functional phage concentration governs bactericidal activity. LC-MS/MS occupies a complementary and distinct position in this landscape, providing a precise, reproducible measure of structural protein concentration that addresses the analytical precision and regulatory rigor that plaque-based and nucleic acid-based methods are not designed to provide. However, the DLA assay remains indispensable as the reference standard for infectivity, and the combination of these approaches offers a more comprehensive characterization of therapeutic phage preparations than any single method alone. This combined approach opens a pathway toward the rigorous PK/PD characterization needed for clinical translation.

A conceptual strength of the signature peptide approach lies in the selection of a target derived from a high-abundance structural capsid protein with a defined copy number per virion. The fixed stoichiometry of capsid proteins provides an intrinsic signal that is directly proportional to phage concentration, offering a stable and structurally invariant quantitation anchor. Unlike tail fiber proteins, which may exhibit variable interactions with bacterial hosts and introduce additional sources of analytical noise, capsid proteins are not subject to the same host-dependent variability, making them well-suited targets for reproducible quantitation across diverse sample types. Furthermore, while PCR-based approaches require independent optimization of amplification conditions for each phage target, a requirement that scales poorly with cocktail complexity, LC-MS/MS enables simultaneous quantitation of multiple phages by selecting unique signature peptides with distinct MRM transitions. This represents a fundamentally different relationship between measured signal and biological target, one that is directly proportional to virion concentration and uniquely suited to the multiplexed quantitation demands of an arbitrary N-phage cocktail.

The practical implications of this approach extend directly to the clinical scenarios where current analytical methods have been unable to fully meet the demands of phage PK/PD characterization. The ELIMINATE trial, which evaluated a six-phage cocktail, illustrates this challenge where quantitation required three high-efficiency host strains and yielded only a composite titer. While a pragmatic solution given available methods, it does not support the individual phage PK/PD characterization needed for cocktail optimization.[27] LC-MS/MS would address this gap by enabling host-independent, simultaneous quantitation of each phage component within a single analytical run. Unlike conventional small molecule drugs, phages must reach the site of infection to interact with susceptible bacteria, meaning that plasma concentrations alone are insufficient to characterize therapeutic exposure. Quantitation at the site of infection, whether in pulmonary tissue, wound fluid, or other relevant compartments, is essential for understanding the exposure-response relationships that govern bactericidal activity.[28] The matrix flexibility and analytical specificity of LC-MS/MS make it particularly well-suited for tissue-level quantitation, where host-dependent methods face compounding sources of variability from endogenous biological components, further limiting already variable quantitative performance.

A significant consideration in evaluating the agreement between LC-MS/MS and DLA-derived concentration estimates is the inherent analytical floor of the plaque assay itself. Plaque count data follows a Poisson distribution, which introduces an irreducible baseline variability that is independent of operator skill or procedural standardization. This Poisson-derived variability establishes a theoretical minimum CV for the DLA that cannot be overcome regardless of how rigorously the assay is performed and was reflected in the CV values observed across stocks in this study. Anderson and colleagues proposed a 0.33 log equivalence threshold as the criterion for agreement between replicates of the DLA, acknowledging that differences within this range are indistinguishable from the natural variability of plaque-based enumeration.[9] When expressed as rRMSE, this threshold corresponds to values of 6.3%, 4.6%, and 3.6% at 10^5^, 10^7^, and 10^9^ PFU/mL respectively. The composite rRMSE of 4.6% observed across the LC-MS/MS and DLA paired measurements falls within this equivalence range, indicating that tandem mass spectrometry is analytically equivalent to the plaque assay within the bounds of what the gold standard method itself can discriminate. This inherent variability floor also has direct implications for experimental design. The imprecision of plaque-based enumeration necessitates replicate measurements to achieve reliable concentration estimates, whereas the analytical precision of mass spectrometry is sufficient to support singlet analysis, making it amenable to high-throughput workflows.

The estimated three copies of the major head protein per virion provide the conversion factor linking peptide molarity to phage particle concentration. The internal consistency of the regression across three independent phage stocks, combined with an intercept that was not significantly different from zero, supports the validity of the proportional relationship between peptide and virion molarity across a 10,000-fold concentration range. It should be noted, however, that PFU-based quantitation selectively enumerates infectious particles capable of completing a lytic cycle on the host isolate and may underestimate total virion concentration if non-infectious or defective particles are present in the lysate preparation. As such, the empirical copy number estimate reflects major head protein stoichiometry relative to infectious particle concentrations. While absolute quantitation using stable isotope-labeled standards is an established approach for protein copy number determination in proteomics, this estimate reflects the analytical response of the detectable tryptic peptide relative to virion molarity. Definitive structural copy number determination would require orthogonal validation by methods such as cryo-electron microscopy or native mass spectrometry. The concordance observed for evolved LUZ19 is a particularly meaningful finding in the context of phage therapy development, where directed evolution has been explored to generate phages with expanded therapeutic host ranges. Following directed evolution using a single-phage Appelmans protocol, LUZ19 demonstrated increased EOP against select clinical isolates that were poorly susceptible to the parental phage. The narrow host range of individual phages has historically limited the utility of phage therapy, driving the development of strategies such as phage cocktails, engineering of receptor binding proteins, and directed evolution to broaden clinical applicability.[29, 30] A fundamental advantage of phage therapy over conventional antibiotics lies in this adaptability, whereas small molecule antibiotics are static entities that cannot adapt as resistance emerges. Phages can be iteratively evolved to overcome emerging resistant strains, offering a renewable therapeutic modality. The concordance of LC-MS/MS-derived concentration estimates with DLA measurements for evolved LUZ19 confirmed conservation of the major head protein, demonstrating that the structural integrity of the quantitation target is maintained after several rounds of replication. This finding supports the broader applicability of the assay to phage variants and suggests that capsid proteins may represent stable quantitation anchors even under selective evolutionary pressure.

Several limitations of this proof-of-concept study warrant explicit acknowledgement. This work was conducted using a single therapeutic phage, LUZ19, and a single tryptic signature peptide derived from the major head protein. While the analytical performance demonstrated here is compelling, generalizability to other phage types, morphologies, and structural proteins has not been established and represents an important avenue for future investigation. The empirical copy number estimate of approximately three is derived from a proportional relationship between detectable peptide molarity and infectious particle concentration as measured by the DLA assay and should be interpreted as an analytical scaling factor rather than a structurally verified copy number. Definitive copy number determination would require orthogonal methods such as cryo-electron microscopy or native mass spectrometry. Additionally, the current assay was developed and validated in phage lysate and has not yet been evaluated in complex biological matrices relevant to clinical practice, such as plasma, urine, or tissue homogenates. Matrix-specific interferences in these sample types may affect assay performance and will require dedicated method development prior to clinical implementation. Finally, the use of PFU/mL as the reference concentration introduces the assumption that infectious particle count approximates total virion concentration, which may not hold if non-infectious or defective particles are present in significant proportions.

Looking forward, several directions present themselves as natural extensions of this work. A logical and immediate next step is the transition toward a fully functional tandem mass spectrometry assay, in which multiple signature peptides derived from the same structural protein, or from multiple structural proteins simultaneously, are monitored within a single analytical run. Such an approach would provide not only redundant quantitative targets that strengthen confidence in concentration estimates, but also a comprehensive analytical fingerprint of the virion that confirms structural integrity, composition, and phage identity in parallel. Agreement between independent peptide-derived concentration estimates would provide strong orthogonal evidence for the reliability of the empirical conversion factor, and internal consistency checks across multiple targets would substantially improve the robustness and clinical readiness of the assay. Translation of the assay to complex biological matrices encountered in clinical practice represents another important next step, as quantitation in clinically relevant compartments would support not only pharmacokinetic characterization of phage distribution but also pharmacodynamic assessment of bactericidal activity at the site of infection. These environments introduce matrix-specific interferences that will require characterization and appropriate sample preparation strategies such as solid-phase extraction. The current assay demonstrated a lower limit of quantitation of approximately 10^5^ PFU/mL using a conventional flow LC system operating at 0.200 mL/min, which falls within a clinically relevant range for therapeutic phage concentrations expected during treatment.[27] Should higher sensitivity be required for specific applications, transitioning to a nano-flow or micro-flow LC configuration would be expected to improve and extend the quantitation range by one to two orders of magnitude. Finally, perhaps the most transformative future direction enabled by this work is the extension to multiplexed enumeration of phage cocktails. By selecting phage-specific signature peptides from each component of a cocktail and monitoring unique MRM transitions simultaneously, individual phage concentrations could be resolved within a single analytical run. This multiplexing capability is fundamentally beyond the reach of existing enumeration methods and would be transformative for cocktail PK characterization and clinical monitoring.

Together, these findings and future directions position mass spectrometry-based signature peptide quantitation not merely as an alternative to existing methods, but as a platform technology capable of supporting the full arc of phage therapy development. From preclinical PK characterization through clinical trial implementation, the analytical rigor and scalability of this approach address long-standing gaps in phage enumeration. Ultimately, the ability to quantify individual phage components with the precision and reproducibility expected in modern drug development represents a meaningful step toward establishing phage therapy as a truly development-ready therapeutic modality.

## Material and Methods

### Materials and Reagents

The tryptic signature peptide EVAELDGQELAR and its stable isotope-labeled extended analog EVAELDGQELARKFD (internal standard), both derived from a LUZ19 capsid protein, were synthesized by Life Technologies Corporation (Carlsbad, CA, USA). HPLC-grade acetonitrile, water, methanol, and acetone, as well as formic acid and acetic acid, were purchased from Fisher Scientific (Fair Lawn, NJ, USA). Trypsin from bovine pancreas, Type T (Sigma-Aldrich, St. Louis, MO, USA), sodium dodecyl sulfate (Thermo Scientific Chemicals, Waltham, MA, USA), iodoacetamide (Thermo Scientific Chemicals, Waltham, MA, USA), and dithiothreitol (Thermo Scientific, Rockford, IL, USA) were used.

### Production of Bacteriophage Stocks

*Pseudomonas aeruginosa* laboratory strain PAO1 was cultured in cation adjusted Mueller-Hinton Broth (Mg^2+^ 25 mg/L and Ca^2+^ 12.5 mg/L) or agar (Becton Dickinson, Sparks, MD, USA) at 37°C. Podovirus LUZ19, classified within the genus *Phikmvvirus*, was isolated on PAO1. Phage propagation and purification were performed as previously described.[8] Briefly, phage lysates were sterilized by 2x high-speed centrifugation and 0.2 μm dead-end filtration.

### Double Layer Agar Assay for Bacteriophage Titration

Phages were quantified by double-aliquot 48-spot serial titration, as previously described.[8] Briefly, PAO1 cultured at OD_600_ 0.2 – 0.3 was lawned and dried over agar medium. Two identical 8-well columns of tenfold serially diluted phage samples were spotted at 4 μL, in sextuplicate. Titrations were incubated at 37°C until plaque forming units (**PFU**) were visible.

### Sample Preparation

A modified surfactant-aided-precipitation/on-pellet-digestion (**SOD**) strategy was employed for sample cleanup.[31] Phage lysate samples were prepared in a solution containing 1.0% sodium dodecyl sulfate and incubated at 90°C for 10 minutes to denature phage capsids. Dithiothreitol (**DTT**) was added to a final concentration of 9.8 mM, followed by incubation at 56°C for 30 minutes to reduce proteins; then iodoacetamide (**IAM**) was added to a final concentration of 58.5 mM and incubated in the dark for 30 minutes at 37°C to prevent reformation of disulfide bonds. One volume of chilled acetone was added to ensure no visible particulate was observed; another three volumes of chilled acetone were then added, followed by 3 hours incubation at −20°C. Post-incubation, samples were centrifuged at 30,279 g at 4°C for 30 minutes, then the supernatant was poured out, washed with one volume of chilled acetone, and the pellet was allowed to air-dry. Ammonium bicarbonate buffer (50 mM, pH = 8.3) was added to the pellet, followed by addition of 2.5 μg of trypsin, and samples were incubated overnight at 37°C to achieve complete digestion. Digestion was terminated by the addition of formic acid to a final concentration of 0.5%.

### Signature Peptide (SP) Selection and Identification

Peptides derived from phage structural proteins were separated on an Acclaim PepMap 100 (75 μm x 15 cm, 2 μm) nanoViper C18 analytical column on an EASY-nLC 1200 system (Thermo Fisher Scientific, Waltham, MA, USA). Separation was achieved through a gradient elution method with mobile phases consisting of 0.1% formic acid in water (Mobile Phase A) and 0.1% formic acid in acetonitrile (Mobile Phase B) at a flow rate of 300 nL/min. The following gradient was applied: 0% B to 2% B at 10 min, 2% B to 40% B at 45 min, 40% B to 100% B at 50 min, followed by a 5-min wash at 100% B. Separated peptides were analyzed using a LTQ Orbitrap XL system (Thermo Fisher Scientific, Waltham, MA, USA). The samples were analyzed in positive mode using the following tune parameters: source voltage 1.5kV, capillary voltage 35V, tube lens 100V. Tandem mass spectrometry was performed using untargeted data-dependent MS^2^ with the top 10 highest abundant precursors selected for fragmentation. Peptide mapping of structural proteins for LUZ19 was performed using the Sequest search algorithm in Proteome Discoverer (version 2.3, Thermo Fisher Scientific, Waltham, MA, USA) and matched against the NCBI FASTA database. A signature peptide was selected from a high-abundance structural capsid protein based on peptide specificity, signal intensity, and chromatographic performance.

### LC-MRM-MS Analysis

Quantitative analysis was performed on a conventional flow LC system coupled to a triple-quadrupole MS. An ExionLC AE was interfaced to a QTRAP 7500+ mass spectrometer (SCIEX, Framingham, MA, USA) via an ESI source. Peptides were separated on an XSelect Premiere HSS T3 column (2.1 mm × 150 mm, 3.5 μm, Waters, Milford, MA, USA). A 15-minute gradient was employed with a flow rate of 0.200 mL/min. Mobile phases consisted of 10% methanol in water containing 0.1% formic acid (Mobile Phase A) and 95% acetonitrile in water containing 0.1% formic acid (Mobile Phase B). The gradient was initiated at 14% B, increasing to 17% B at 6.5 min, then to 23% B at 8 min, followed by a step to 95% B from 8.1 to 10.5 min, and re-equilibrated at 14% B from 10.6 to 15 min. The column temperature was 40°C. The spray voltage was 2.5 kV, and the capillary temperature was 415°C. Quantitation was performed using multiple reaction monitoring of the transitions *m/z* 665.3 → 673.5 [(M+2H)^2+^ → y_6_], *m/z* 665.3 → 788.5 [(M+2H)^2+^ → y_7_], and *m/z* 665.3 → 901.5 [(M+2H)^2+^ → y_8_] for the analyte, and *m/z* 670.5 → 798.2 and *m/z* 670.5 → 1040.3 for the internal standard. The optimized collision energy was set to 32 V, the Q0 dissociation was set to 70 V, and dwell time was 150 ms for each transition. Data acquisition and processing were performed using SCIEX OS (version 3.3.0, SCIEX, Framingham, MA, USA) and statistical analyses were performed using R (version 4.4.2, R Foundation for Statistical Computing, Vienna, Austria).

### Preparation of Calibrators and Establishment of Calibration

The extended analog of the tryptic signature peptide EVAELDGQELAR, derived from a LUZ19 capsid protein (EVAELDGQELARKFD), was prepared as a stable isotope-labeled peptide analog incorporating [^13^C_6_, ^15^N_4_]-arginine and used as an internal standard (**IS**) at a fixed concentration of 1.2 pg/mL. Calibrator solutions of the tryptic signature peptide were prepared at 0.008, 0.08, 0.8, 8, 50, and 80 pg/mL.[32] The internal standard was used to correct for variability introduced by enzymatic digestion and by instrument response. The purities of the extended and isotope-labeled peptide analogs were validated by quantitative amino acid analysis (**AAA**). Quantitation was performed using peak area ratios of the signature peptide relative to the isotope-labeled IS. Linear regression with a 1/*x*^2^ weighting factor was applied to construct the calibration curve.

### Copy Number Determination

Three independent phage lysate stocks were prepared by 1:10 serial dilutions over a concentration range of 10^5^ to 10^9^ PFU/mL using the SOD strategy. Peak area ratios of the surrogate peptide derived from digested phage lysate samples were plotted against a calibration curve to derive peptide concentrations, which were subsequently converted to femtomolar units (Equation 1). Nominal phage concentrations were independently converted from PFU/mL to femtomolar units using Avogadro’s number (Equation 2), with the caveat that PFU/mL quantifies infectious particles capable of completing a lytic cycle on the host isolate and may underestimate total virion concentration. Weighted Deming regression was performed in R to relate peptide molarity to virion molarity, with the slope of the regression used as an empirical estimate of copy number per virion.


Equation 1: $$fM=\frac{C_{peptide}\;(pg∕mL)\cdot 10^{6}}{MW}$$

Equation 2: $$fM=\frac{C_{phage}\;(PFU∕mL)\cdot 10^{18}}{6.022\cdot 10^{23}\;mol^{-1}}$$

### Phage Directed Evolution and Efficiency of Plating (EOP)

Nine clinical *Pseudomonas aeruginosa* isolates (AR-0236, AR-0240, AR-0245, AR-0246, AR-0250, AR-0254, AR-0257, AR-0259, and AR-0270) obtained from the CDC/FDA Antibiotic Resistance (**AR**) Isolate Bank were used for directed evolution and EOP assessment. Directed evolution of LUZ19 was performed using a modified single-phage Appelmans protocol.[33] Using a 96-well plate format, serial 10-fold dilutions of LUZ19 were prepared across each row, with each row corresponding to a distinct clinical isolate. Following overnight incubation at 37°C, wells exhibiting clearing and the first turbid transition well were pooled across all rows into a single master lysate, clarified by centrifugation, and filtered-sterilized. This evolved phage lysate was then used as the input for the subsequent rounds of selection, with the process repeated iteratively to promote host range expansion. EOP was calculated as the ratio of PFU/mL obtained on each clinical isolate relative to the PFU/mL obtained on the reference strain PAO1, assessed at Day 0 and following directed evolution using the DLA method.[34]

### Validation and Evaluation of Quantitative Performance

Method performance was evaluated using three independent analytical runs on separate days to assess intra- and inter-day variability. Quality control (QC) samples of the tryptic peptide EVAELDGQELAR, prepared at 0.006, 0.4, and 40 pg/mL, were evaluated alongside each calibration curve to assess accuracy and precision across runs. To evaluate the accuracy of the empirical copy number conversion factor, phage lysate QC samples were prepared from both unmodified LUZ19 and evolved LUZ19 at approximately 10^5^, 10^7^, and 10^9^ PFU/mL. Aliquots of these QC samples were assessed in triplicate for each of the three different days. Phage concentrations estimated from the MS method using the empirical conversion factor (Equation 3) were compared to nominal concentrations determined by DLA, with method agreement assessed using relative root mean square error (rRMSE) and relative bias (rBias).[26] Conservation of the capsid protein in evolved LUZ19 was confirmed by the concordance of MS-derived concentration estimates with DLA measurements.


Equation 3: $$C_{phage}\;(PFU∕L)=\frac{C_{peptide}(mol∕L)\cdot 6.022\cdot 10^{23}\;mol^{-1}}{copy\;number}$$

## Figures and Tables

**Table 1:** Structural proteins and sequence coverage.

Description	No. of Peptides	Coverage (%)	MW (kDa)
YP_001671977.1 major head protein	15	55	37.6
YP_001671995.1 hypothetical protein	2	46	7.8
YP_001671994.1 minor head protein	1	25	8.7
YP_001671965.1 hypothetical protein	1	2	36.8

**Table 2:** Back-calculated accuracy of calibration standards across three independent analytical runs.

Day	Nominal Concentration (pg/mL)	Calculated Concentration (pg/mL)	Mean Accuracy (%)	Bias (%)
1	80	82.9	103.6	3.6
1	50	51.6	103.2	3.2
1	8	7.97	99.7	−0.3
1	0.8	0.785	98.1	−1.9
1	0.08	0.0759	94.9	−5.1
1	0.008	0.00804	100.5	0.5
2	80	81.3	101.6	1.6
2	50	52.1	104.2	4.2
2	8	8.15	101.8	1.8
2	0.8	0.777	97.1	−2.9
2	0.08	0.0757	94.6	−5.4
2	0.008	0.00805	100.6	0.6
3	80	79.7	99.6	−0.4
3	50	51.4	102.8	2.8
3	8	7.74	96.8	−3.2
3	0.8	0.865	108.2	8.2
3	0.08	0.0735	91.8	−8.2
3	0.008	0.00806	100.7	0.7

**Table 3:** Intra-day precision and accuracy.

Day	QC Level	Nominal Concentration (pg/mL)	n	Mean Calculated Concentration (pg/mL)	CV (%)	Mean Accuracy (%)	Bias (%)
1	H	40	3	38.5	1.3	96.3	−3.7
1	M	0.4	3	0.349	1.9	87.2	−12.8
1	L	0.006	3	0.00554	2.7	92.4	−7.6
2	H	40	3	35.3	2.0	88.2	11.8
2	M	0.4	3	0.321	0.5	80.2	−19.8
2	L	0.006	3	0.00517	9.8	86.1	−13.9
3	H	40	3	41.6	0.8	104.0	4.0
3	M	0.4	3	0.370	0.6	92.5	−7.5
3	L	0.006	3	0.00625	2.5	104.1	4.1

**Table 4:** Inter-day precision and accuracy.

QC Level	Nominal Concentration (pg/mL)	n	Mean Calculated Concentration (pg/mL)	CV (%)	Mean Accuracy (%)	Bias (%)
H	40	9	38.5	7.2	96.2	−3.8
M	0.4	9	0.347	6.3	86.6	−13.4
L	0.006	9	0.00565	9.7	94.2	−5.8

**Table 5:** Efficiency of plating of LUZ19 after directed evolution.

Bacterial isolate	Day 0	Day 10
PAO1	1.0000	1.0000
AR-0236	0.5582	1.3750
AR-0240	0.0000	0.0000
AR-0245	0.0000	0.0000
AR-0246	0.0000	0.0000
AR-0250	0.0000	0.0000
AR-0254	0.0000	0.0021
AR-0257	0.0001	0.3250
AR-0259	0.0000	0.0000
AR-0270	1.0702	1.2500

## Data Availability

Mass spectrometry data have been deposited in the MassIVE repository under accession number PXD077596 and are publicly accessible at doi:10.25345/C5RX93T21. All other data supporting the findings of this study are included in the article or are available from the corresponding author upon reasonable request.
